# The phage growth limitation system in *Streptomyces coelicolor* A(3)2 is a toxin/antitoxin system, comprising enzymes with DNA methyltransferase, protein kinase and ATPase activity

**DOI:** 10.1016/j.virol.2014.12.036

**Published:** 2015-03

**Authors:** Paul A. Hoskisson, Paul Sumby, Margaret C.M. Smith

**Affiliations:** a Department of Molecular and Cell Biology, University of Aberdeen, Institute of Medical Science, Foresterhill, Aberdeen AB25 2ZD, UK; b Department of Genetics, University of Nottingham, Queens Medical Centre, Nottingham NG7 2UH, UK

**Keywords:** Bacteriophage, Streptomyces, Immunity, Defence, Restriction, Modification

## Abstract

The phage growth limitation system of *Streptomyces coelicolor* A3(2) is an unusual bacteriophage defence mechanism. Progeny ϕC31 phage from an initial infection are thought to be modified such that subsequent infections are attenuated in a Pgl^+^ host but normal in a Pgl^−^ strain. Earlier work identified four genes required for phage resistance by Pgl. Here we demonstrate that Pgl is an elaborate and novel phage restriction system that, in part, comprises a toxin/antitoxin system where PglX, a DNA methyltransferase is toxic in the absence of a functional PglZ. In addition, the ATPase activity of PglY and a protein kinase activity in PglW are shown to be essential for phage resistance by Pgl. We conclude that on infection of a Pgl^+^ cell by bacteriophage ϕC31, PglW transduces a signal, probably *via* phosphorylation, to other Pgl proteins resulting in the activation of the DNA methyltransferase, PglX and this leads to phage restriction.

## Highlights

• The phage growth limitation (Pgl) system is a novel and elaborate mechanism of phage resistance.
• The system comprises, in part, a toxin/antitoxin system.
• The toxic protein, PglX, has DNA methyltransferase activity.
• The antitoxin is conferred by PglZ.
• Proteins with ATPase (PglY) and kinase (PglW) activities are also required for resistance.

## Introduction

**Fig. 1:**
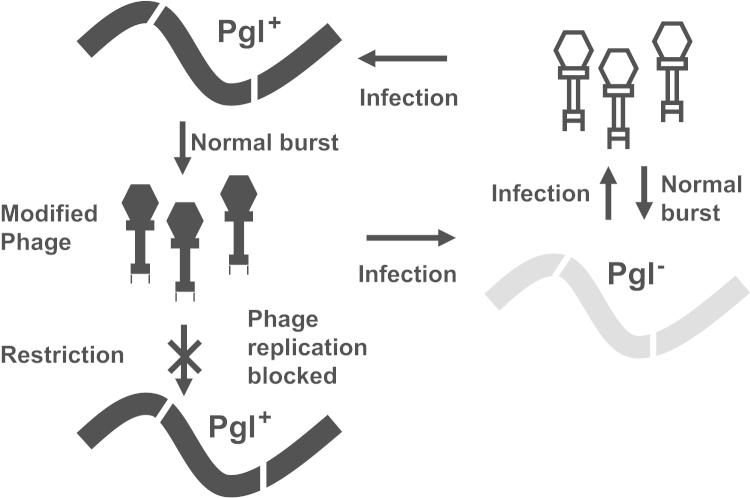
A schematic of the Pgl phenotype, based on the model of Chinenova et al. (1982). The white-filled, phage-like particles represent unmodified phage, and the black-filled, phage-like particles represent modified phage. Pgl^+^ (black) and Pgl^−^ (grey) host mycelia are represented as curved lines.

Bacteria have evolved a plethora of diverse mechanisms to evade killing by bacteriophages. The mechanisms can act at any stage of the phage life cycle from preventing phage adsorption right through to inhibition of cell lysis and release of progeny phage (Hoskisson and Smith, 2007). The phage growth limitation (Pgl) system of *Streptomyces coelicolor* confers resistance against the temperate bacteriophage ϕC31 and its homoimmune relatives (Chinenova et al., 1982; Laity et al., 1993). This system is called ‘growth limitation’ as phage infecting a Pgl^+^ strain for the first time undergoes a normal single burst to produce progeny phage but the progeny is attenuated for growth in a second round of infection. The progeny is however able to form normal plaques on a Pgl^−^ host (Fig. 1). The mechanistic explanation of the Pgl phenotype proposed by Chinenova et al. (1982) is that during the initial round of infection the progeny phage are modified and then restricted in the second round of infection. This system differs fundamentally from typical R–M systems, where, if the phage DNA becomes modified, there can be an escape from restriction and rapid spread of infection through sensitive bacteria. In Pgl, however, even if modification fails during the first burst, it is likely that it will occur in subsequent cycles thereby severely limiting spread of infection (Fig. 1; Sumby and Smith, 2002). Furthermore Pgl may confer an added advantage to a clonal population as being Pgl^+^ amplifies phage that might infect and kill phage-sensitive competitors.

**Fig. 2:**
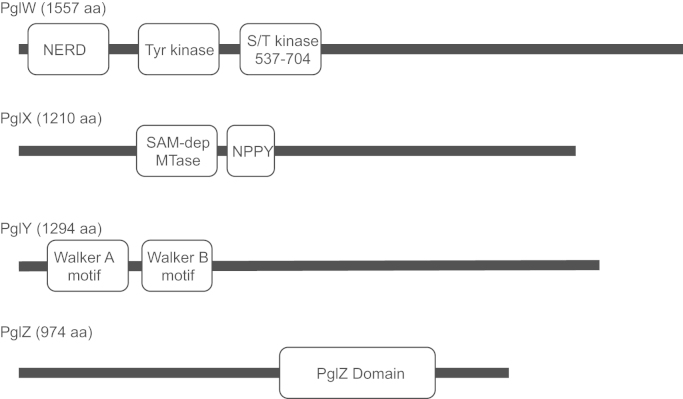
Predicted functional motifs in the Pgl proteins as revealed by Prosite or InterProScan searching (Apweiler et al., 2000; Hulo et al., 2008). PglW contains three predicted motifs; NERD (Nuclease-related domain; 12–130 aa); an atypical tyrosine kinase domain (195–490 aa); a Hanks-like serine/threonine protein kinase domain (537–704 aa). PglX contains a SAM-dependant methyltransferase motif (209–748 aa) and an N6-adenine methyltransferase motif (NPPY; 378–381 aa). PglY contains Walker A and Walker B motifs (75–82 aa and 285–289 aa, respectively). PglZ contains a predicted alkaline phosphatase fold (525–792 aa), annotated as ‘PglZ domain’ in Pfam (PF08665).

Previous work has identified four genes, located in two operons (*pglWX* and *pglYZ*) 6 kbp apart, required for Pgl in *S. coelicolor* (Bedford et al., 1995; Laity et al., 1993; Sumby and Smith, 2002). Bioinformatic searches on the products of the *pgl* genes revealed several protein motifs (Fig. 2). The product of PglX is predicted to bind AdoMet and has an N^6^-adenine DNA methyltransferase motif, PglW is a putative serine/threonine protein kinase that contains a typical Hanks-like protein kinase domain, PglY possesses putative Walker A and Walker B motifs for the binding and hydrolysis of ATP/GTP and PglZ is predicted to have a conserved alkaline phosphatase-like fold (Pfam: PF08665). While the predicted function of PglX as a DNA methyltransferase fits well with the proposed mechanism by Chinenova et al. (1982) the Pgl system appears to involve additional activities that are novel to R–M systems, not least the putative kinase activity of PglW.

Transcriptional analysis of *pgl* genes indicates that both operons are transcribed, even in the absence of phage infection (Bedford et al., 1995; Sumby and Smith, 2002). While transcriptional up-regulation of these genes is plausible during infection, we cannot rule out a role for the control of protein function through phosphorylation given that PglW is a predicted protein kinase. Indeed it has been shown that two isoforms of PglZ were detected with different isoelectric points in 2D-PAGE gels, suggesting that this protein could be post-translationally modified (Hesketh et al., 2002).

Another feature of the Pgl system is that it is subject to high frequency phase variation in which a Pgl^+^ strain gives rise to a Pgl^−^ strain with a frequency of 10^−2^ to 10^−3^ and Pgl^−^ to Pgl^+^ with a frequency of 10^−3^ to 10^−4^ (Chinenova et al., 1982; Laity et al., 1993). The phase variation has been attributed to a variation in length of a G tract in *pglX* (Sumby and Smith, 2003). Switching the phage resistance on and off may help to ease the strong selection within the phage population to mount a counter defence (Bayliss et al., 2006).

Here we set out to test the major bioinformatic predictions of Pgl protein functions. In the process we identified a toxin/antitoxin system comprising a toxic PglX protein, shown to be a DNA methyltransferase, and an antitoxin, PglZ. We also demonstrate that the protein kinase activity of PglW, and the Walker A motif of PglY are required for a functional Pgl system. We present a model as to how the Pgl proteins might confer phage resistance in *S. coelicolor* through an elaborate and novel R–M-like system.

## Results

We set out to test whether mutations in the predicted functional motifs of the Pgl proteins were required for the phage defence phenotype (Fig. 2). The strategy used was to generate knock-out or null mutations in each *pgl* gene and then complement these null mutants with either wild type (wt) or mutant alleles introduced ectopically into the chromosome using the ϕC31 *int*/*attP* system (Bierman et al., 1992). Where possible we obtained further proof of Pgl protein function by heterologous expression of Pgl protein in *Escherichia coli* and biochemical assay of the partially purified extracts.

### PglX is a DNA methyltransferase

**Fig. 3:**
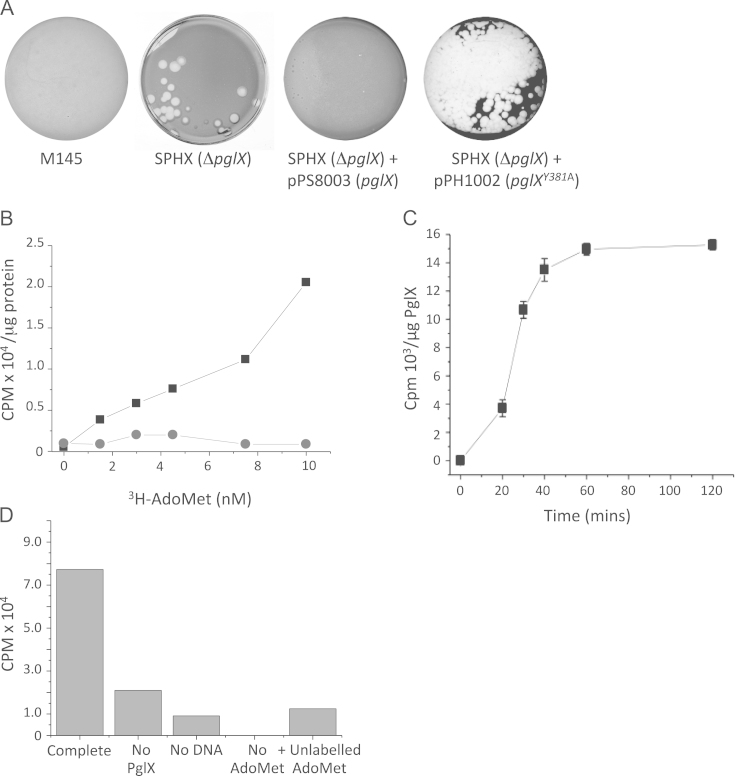
PglX methyltransferase activity is required for the Pgl phenotype. (A) SPHX (*ΔpglX*) was challenged with 3×10^2^ pfu/ml of ϕC31*c*Δ25, grown on J1929 (*pglY*^*−*^) Bedford et al. (1995). Wild type *Streptomyces coelicolor* M145 (*pglX*^*+*^), SPHX pPS8003 (*ΔpglX*/*pglX*^*+*^) and SPHX pPH1002 (*ΔpglX*/*pglX*^*Y381A*^) were challenged with 1×10^5^ pfu ml^−1^ ϕC31*c*Δ25. (B) Methyltransferase activity of heterologously expressed PglX–His_6_ (squares) and PglX^Y381A^–His_6_ (circles) using ^3^H-AdoMet as the methyl donor to ϕC31*c*Δ25 DNA isolated from J1929 (*pglY*^*−*^) Bedford et al. (1995). Data are the mean of two replicate experiments. (C) Time dependent methyltransferase activity of PglX–His_6_ on ϕC31*c*Δ25 DNA isolated from J1929 (*pglY*^*−*^). Data are the mean of three replicate experiments and error bars represent the SD of the data. (D) Dependence of methyltransferase activity of PglX–His_6_ on protein, ϕC31*c*Δ25 DNA and competition by addition of 10 nM unlabelled AdoMet to indicate specificity of activity (data are the mean of two replicate experiments).

Bioinformatic analysis of the PglX sequence suggested the presence of an N^6^-adenine methyltransferase motif (NPPY) at 378–381 aa (Roth et al., 1998; Scavetta et al., 2000; Fig. 2). A *ΔpglX* null mutant, SPHX, was constructed using REDIRECT technology that involves recombineering of the kanamycin-resistant cosmid SCIF2, replacing the *pglX* coding sequence (SCO6627) with an apramycin resistance gene. The apramycin resistance gene is flanked by *loxP* sites and also contains an origin of transfer (*oriT*) to enable conjugation of the cosmid into the Pgl^+^ *S coelicolor* strain, M145 (Gust et al., 2003; Redenbach et al., 1996). Double crossovers that retain the apramycin marker but had lost the kanamycin resistance marker were then infected with Cre-phage in a transient infection to remove the apramycin resistance marker (Khodakaramian et al., 2006). SPHX was sensitive to ϕC31 and could be complemented by the introduction of pPS8003 encoding a His-tagged version of PglX (Fig. 3A; Sumby and Smith, 2002).

To test whether the putative methyltransferase domain of PglX was necessary for Pgl function, the tyrosine residue (Y381) of the conserved NPPY motif was targeted by site directed mutagenesis (Sumby and Smith, 2002). This tyrosine residue in other methyltransferases is structurally essential for catalysis where it is required for flipping out the target base from the DNA double helix prior to methylation (Roth et al., 1998). The resulting plasmid, pPH1002 (encoding PglX^Y381A^–His_6_) was conjugated into SPHX (*ΔpglX*) to test *in vivo* activity of the mutated *pglX* allele. PglX^Y381A^ did not restore phage resistance to SPHX suggesting that the putative methyltransferase motif is essential for the Pgl system (Fig. 3A).

To assay DNA methyltransferase activity of PglX, attempts were made to express a C-terminally His-tagged PglX fusion in *E. coli* and to enrich extracts by affinity chromatography. The 136 kDa PglX–His_6_ from *E. coli* was barely detectable using a 6× His antibody in Western blots of the enriched proteins (data not shown). Nevertheless, low levels of methyltransferase activity using ϕC31 DNA as a substrate were observed (Fig. 3B–D). In a time course of methyltransferase activity, incorporation of ^3^H-methyl groups into TCA precipitable material increase over the period of 60 min (Fig. 3C) and the level of incorporation was dependent on the amount of protein added (data not shown). An extract of PglX^Y381A^–His_6_ was prepared in an identical procedure to the expression and enrichment of PglX–His_6_, but methyltransferase activity was almost undetectable (Fig. 3B), ruling out the possibility that the observed activity was due to endogenous *E. coli* enzymes. Further controls confirmed that incorporation of label into TCA precipitable material was dependent on DNA addition and could be competed by addition of unlabelled AdoMet (Fig. 3D). These data strongly suggest that PglX can methylate DNA in an *in vitro* assay.

### A *pglZ* null mutation is lethal in strains with a wild type *pglX* gene

**Fig. 4:**
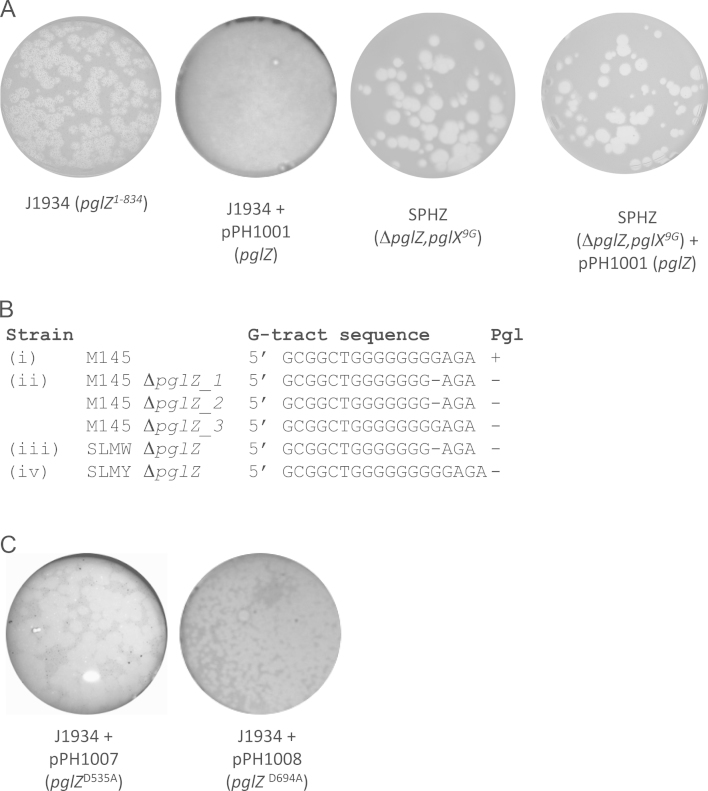
Residues in the conserved PglZ domain are required for the Pgl phenotype. (A) Strains (indicated) were challenged with 1×10^4^ to 1×10^5^ pfu ml^−1^ of ϕC31*c*Δ25 grown on J1929 (*pglY*^*−*^). (B) Alignment of G-tract in *pglX* in M145, Δ*pglZ* null mutants indicating the expansion or contraction of the G-tract in these strains. DNA sequences starting at sequence coordinate 7353977 from (i) the reference parent strain M145 (Genbank accession: NC_003888), (ii) three independent *ΔpglZ* exconjugants from M145, (iii) a *ΔpglZ* exconjugant from SPHW, (iv) a *ΔpglZ* exconjugant from SLMY. (C) Mutagenesis of conserved residues in PglZ fail to complement the *pglZ* mutant J1934. Strains (indicated) were challenged with 1×10^4^ to 1×10^5^ pfu ml^−1^ of ϕC31*c*Δ25 grown on J1929 (*pglY*^*−*^).

*In silico* predictions on the function of PglZ are limited to a region (527–704 aa) annotated as a ‘PglZ domain’ (Pfam: PF08665), which falls within a family of proteins called the ‘alkaline phosphatase clan’ (Finn et al., 2010; Fig. 2). We attempted to create a knockout mutant of *pglZ* in M145. REDIRECT technology was used to replace the *pglZ* ORF in the cosmid SC4G2, with the apramycin marker generating a cosmid SC4G2:*ΔpglZ::apra*. When this cosmid was introduced into M145 by conjugation, an extremely low frequency of double recombinants (kanamycin sensitive, apramycin resistant) was obtained (Table 2). Three putative M145*::*Δ*pglZ* colonies were propagated and were found to be phage sensitive as expected. However, introduction of *pglZ–His*_*6*_ encoded by the integrating plasmid, pPH1001, was unable to complement the phage sensitive phenotype of any of these recombinants (Fig. 4A). To demonstrate that pPH1001 was able to complement a *pglZ*^*−*^ defective allele, the plasmid was introduced into J1934, a Pgl^−^ strain constructed by Bedford et al. (1995) by insertional inactivation resulting in the deletion of the 3′ end of *pglZ* encoding the C-terminal 130 amino acids (referred to as *pglZ*^*1-834*^). The plasmid pPH1001 complemented the *pglZ*^*1-834*^ allele J1934 to give phage resistance (Fig. 4A). These data suggest that the M145*::ΔpglZ* strains made by the REDIRECT approach had acquired a secondary mutation, possibly in one of the other *pgl* genes.

The most likely site for a second site mutation is the G-tract present within *pglX* that had been shown previously to inactivate Pgl (Sumby and Smith, 2003). Sequencing through the G-tract indicated that in two of the three M145*::*Δ*pglZ* strains the number of G residues had contracted (from 8 to 7 G nucleotides; Fig. 4B (i) and (ii)), while in the third strain the G-tract was as for the Pgl^+^ wild type M145 (8 G nucleotides; Fig. 4B (i) and (ii)), however this strain was also not complemented by pPH1001 indicating that the secondary mutation must be elsewhere in the genes required for Pgl. The provision (*via* integration of pPH1001) of a second copy of *pglZ* in the Pgl^+^ wild type strain M145 enabled the disruption of the native *pglZ* at a frequency of recombination that is typical when an inessential gene is targeted, such as *pglW* by the cosmid SCIF2:*ΔpglW::apra* (Table 2; Gust et al., 2003; Khodakaramian et al., 2006).

To confirm the essentiality of an intact *pglZ*, we first performed alignments of related PglZ proteins from the sequence databases to identify conserved residues that we could target for mutagenesis. Alignment of PglZ homologues indicated the presence of conserved residues D535 and D694. These residues fall within the predicted alkaline phosphatase fold (525–792 aa; Fig. 2; Quevillon et al., 2005). D535 and D694 in PglZ were both changed to alanine by site directed mutagenesis in the plasmid pPS5045 and the mutant alleles were subcloned into the integrating vector pIJ6902 (Huang et al., 2005) to generate pPH1007 (encoding PglZ^D535A^–His_6_) and pPH1008 (encoding PglZ^D694A^–His_6_). After conjugation of pPH1007 and pPH1008 into J1934, containing the *pglZ*^*1-834*^ allele, the exconjugants failed to complement the Pgl^−^ phenotype indicating that both *pglZ*^*D535A*^ and *pglZ*^*D694A*^ alleles were defective (Fig. 4C). Plasmid, pPH1008 (encoding PglZ^D694A^–His_6_), was then conjugated into M145 and exconjugants were used as recipients for the SC4G2*:ΔpglZ::apra* cosmid, but the formation of the *pglZ* knockout strains was again prevented (Table 2). These experiments confirmed that the *pglZ*^*D694A*^ allele was unable to confer antitoxin activity in the presence of an otherwise intact Pgl system.

These observations indicate that PglZ may be interacting with PglX to inhibit a toxic activity. To test whether mutations in other *pgl* genes would permit the disruption of *pglZ*, the cosmid, SC4G2*:ΔpglZ::apra*, was conjugated into strains SPHX (Δ*pglX*), SPHW (*ΔpglW*) and SLMY (*ΔpglY*) (Tables 1 and 2). Deletions of *pglZ* were obtained at low frequency, similar to that observed for the deletion of *pglZ* in a wild type background, except in SPHX (Δ*pglX*), where deletion of *pglZ* was obtained at the normal frequency (Table 2). The G tracts in one SPHW::*ΔpglZ* and one SLMY*::ΔpglZ* strains were sequenced and they had suffered a contraction and an expansion, respectively (Fig. 4B (iii) and (iv)). These data are indicative of a specific suppression of a toxic activity of PglX by PglZ. We hypothesise that the *pglZ*^*1-834*^ allele in J1934 might retain the protective activity required against an intact *pglX*.

### PglW has kinase activity

If PglZ prevents the toxic activity of PglX, then the presence of a signalling mechanism that regulates Pgl activity would enable tight control of the system and prevent unwanted toxicity. Bioinformatic analysis of the PglW sequence reveals the presence of two putative protein kinase domains; a tyrosine kinase domain at 195–490 aa (Prosite; PS00109) and a putative serine/threonine protein kinase (STPK, Hanks-type) domain (Prosite; PS00108) at 530–816 aa (Fig. 2). However only the putative STPK domain has a predicted ATP binding site that includes the central core of the catalytic loop and the invariant lysine. This residue (K677; Sumby and Smith, 2002) was targeted by asymmetric PCR mutagenesis. It has previously been shown that the equivalent residue to K677 is absolutely conserved in the ATP binding domain of such proteins, and it is believed to be essential for autophosphorylation and the phosphotransfer reaction (Hanks and Hunter, 1995; Hanks et al., 1988; Young et al., 2003).

**Fig. 5:**
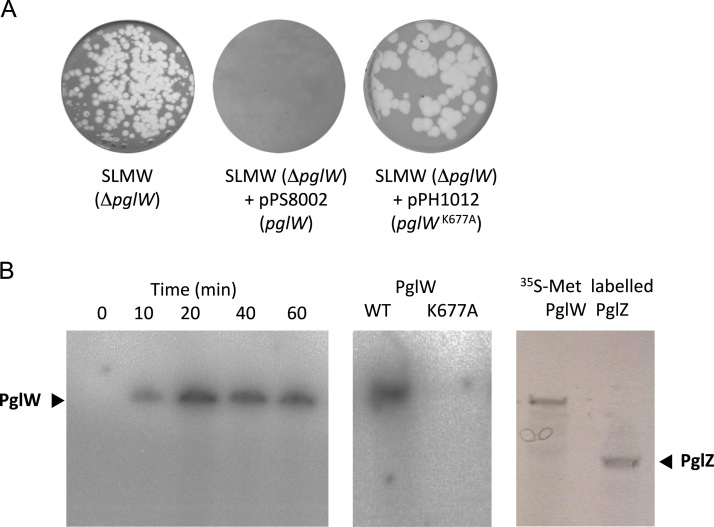
The conserved lysine in the STPK domain of PglW is required for Pgl function. (A) Strains (indicated) were challenged with 1×10^4^ to 1×10^5^ pfu ml^−1^ of ϕC31*c*Δ25 harvested following J1929 infection (PglY^−^). (B) *In vitro* phosphorylation of PglW. (i) Autoradiograph showing a time course of autophosphorylation of *in vitro*-synthesized PglW incubated over 60 min in the presence of [γ-^32^P] ATP. (ii) Autophosphorylation of *in vitro*-synthesized PglW and lack of autophosphorylation activity of PglW^K677A^ in the presence of [γ-^32^P] ATP for 60 min. (iii) Confirmation of *in vitro* expression of PglW and PglZ by incorporation of ^35^S-methionine labelling to synthesised protein, followed by SDS–PAGE and autoradiography.

The resulting plasmid pPH1012 (encoding PglW^K677A^–His_6_) was conjugated into SLMW (*ΔpglW*) to test whether the *pglW*^*K677A*^*–His*_*6*_ allele could complement for the deletion of *pglW* and restore phage resistance. The PglW^K677A^–His_6_ mutation resulted in a phage-sensitive phenotype, indicating that the putative Hanks-like protein kinase domain is both functional and required for a Pgl^+^ phenotype (Fig. 5A).

The gene encoding PglW–His_6_ was cloned into the *E. coli* expression vector pT7-7 to generate pPS5012 and introduced into *E. coli* BL21 DE3(pLysS). No expression of PglW–His_6_ was detected. Examination of the codon usage at the start of the *pglW* ORF showed the presence of several codons that are rarely used in *E. coli*. The first 19 codons were therefore optimised for expression in *E. coli* generating the expression plasmid pPS5025. The frequency of transformation of *E. coli* BL21 DE3 (pLysS) by pPS5025 was very low and expression trials of the few colonies that were obtained indicated the presence of insoluble and truncated proteins (data not shown). It seems likely that PglW–His_6_ is toxic in *E. coli* BL21 and only plasmids that have suffered mutations can be established, explaining the poor transformation frequencies. At this stage we do not understand the basis for the toxicity of PglW although PglW has a putative, but as yet uncharacterised, N-terminal nuclease-related domain (NERD) motif (Fig. 2).

The plasmid pPS5025 was used as a template in an *in vitro* expression system to generate sufficient full length PglW–His_6_ to test for autokinase activity (Molle et al., 2003). Incubation of PglW–His_6_ with [γ-^32^P] ATP resulted in autokinase activity of the protein, resulting in incorporation of radioactive phosphate (Fig. 5B). A control reaction, incorporating ^35^S-methionine into *in vitro* expressed PglW–His_6_ showed that the autophosphorylating band had the same mobility as PglW–His_6_ (Fig. 5B). The K677A mutant allele of PglW was also tested in the same assay for its ability to autophosphorylate; however, no radioactive signal could be detected, indicating that this residue is essential for the autophosphorylation reaction (Fig. 5B). These data are consistent with PglW forming part of a signal transduction system that senses the modification state of phage DNA during infection of a Pgl^+^ cell.

### PglY has ATPase activity

Bioinformatic analysis of the PglY sequence suggested the presence of a Walker A (ATPase) motif (GSFGSGKS) at 75–82 aa (Bedford et al., 1995). It has previously been shown that these motifs are involved in nucleotide binding, and is found in many protein families (Karata et al., 1999; Ramakrishnan et al., 2002). We decided to test whether the ATPase motif is required for resistance to ϕC31 as a means to determine the role PglY might have in the Pgl phenotype.

**Fig. 6:**
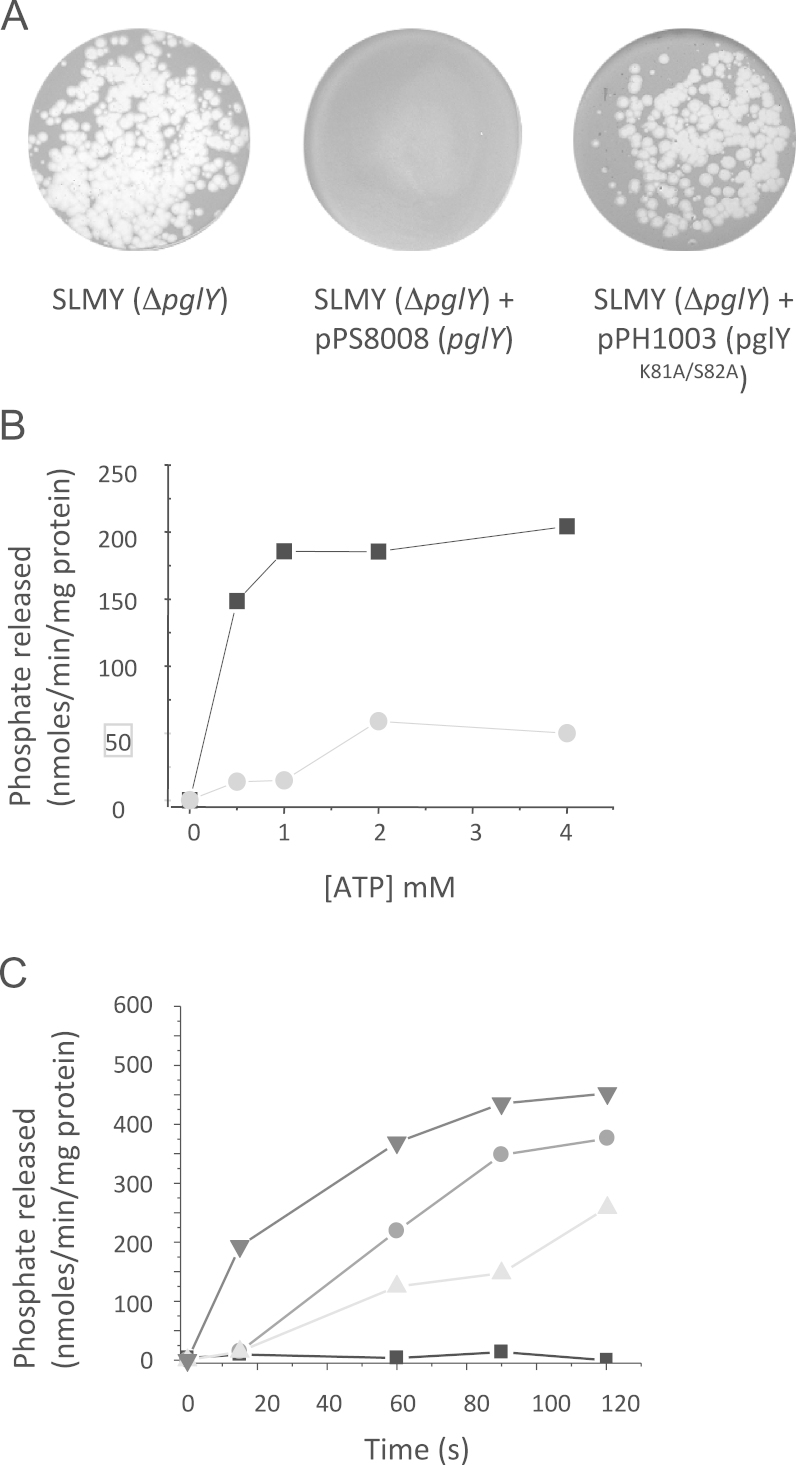
The Walker A motif of PglY is required for Pgl function. (A) Strains (indicated) were challenged with 1×10^5^ pfu ml^−1^ of ϕC31*c*Δ25 harvested following J1929 infection (PglY^−^). (B) Release of P*i* from ATP as a function of the PglY ATPase domain in wild type PglY and the PglY^K81A/S82A^ mutant. Wild-type PglY (squares) and PglY^K81A/S82A^ (circles). Data are mean of 3 replicate experiments. (C) Hydrolysis of ATP in the presence of ADP-NP. 4 mM ATP (black triangles; 3 mM ATP, 1 mM ATP-NP (circles); 2 mM ATP, 2 mM ATP-NP (grey triangles); 1 mM ATP, 3 mM ATP-NP (squares). Data are mean of three replicate experiments.

The essential consecutive lysine and serine residues (K81, S82) were targeted by site directed mutagenesis in the plasmid pPS8008, which encodes PglY (Sumby and Smith, 2002). The resulting plasmid pPH1003 (encoding His_6_–PglY^K81A/S82A^) was conjugated into SLMY (*ΔpglY*). The K81A/S82A double substitution in PglY resulted in a phage-sensitive phenotype suggesting that ATP binding and/or hydrolysis is essential for conferring resistance to bacteriophage infection *via* the Pgl system (Fig. 6A).

N-terminally His-tagged PglY was expressed in *E. coli* and purified by nickel affinity chromatography. A single band was observed at ~160 kDa; this band was excised and subjected to peptide mass fingerprinting by MALDI–TOF mass spectrometry and positively identified as PglY (top hit against the NCBInr protein database). The same approach was subsequently used for purifying His_6_–PglY^K81A/S82A^. The His-tagged wild-type and mutant PglY proteins were tested for their ability to bind and hydrolyse ATP. His_6_–PglY was found to hydrolyse ATP with a substrate affinity (*K*_M_) for ATP of 0.5 mM, the *K*_M_ of the mutant protein His_6_–PglY^K81A/S82A^ was 200-fold higher (100 mM; Fig. 6B). These data indicate that PglY is a functional ATPase. PglY nucleotidase activity was specific for ATP, and was found not to hydrolyse the other nucleotides tested (AMP, ADP, GMP, GDP, and GTP; data not shown). The non-metabolisable ATP analogue, ADP-NP was tested for its ability to inhibit the activity of PglY *in vitro*. The addition of equivalent molar amounts of ADP-NP and ATP, and subsequent 2-fold dilutions of ADP-NP in each assay resulted in a severely inhibited rate of hydrolysis of ATP, indicative of ADP-NP binding and inhibition of activity.

Taken together these data indicate an essential role for the ATPase activity of PglY in the Pgl system.

## Discussion

Previous work describes the phenotype of Pgl in which an infection of *S. coelicolor* Pgl^+^ by phage ϕC31 undergoes a single burst but subsequent infections by progeny phage of *S. coelicolor* Pgl^+^ are attenuated (Bedford et al., 1995; Laity et al., 1993). A logical mechanistic explanation of these observations is that the phage is modified in the first infection but the second infection is restricted. Modified phage is able to proceed through a normal infection cycle in a Pgl^−^ strain. In this work we demonstrated that PglX is indeed a DNA methyltransferase, as predicted by the bioinformatics searches. A strong genetic interaction between *pglX* and *pglZ* implies that PglX is toxic and that toxicity is suppressed in strains that are *pglZ*^*+*^ or contain the truncated *pglZ*^1-834^ allele in J1934. This interaction resembles a toxin/antitoxin system. Many phage resistance mechanisms rely on some type of toxin/antitoxin pair to enable phage restriction and host immunity (Makarova et al., 2013). Examples include the restriction–modification systems where host protection from the endonuclease is usually conferred by DNA modification. However *S. coelicolor* is unusual as it is known to contain methyl-specific endonucleases (Gonzalez-Ceron et al., 2009; MacNeil, 1988). It is therefore feasible that it is the DNA methyltransferase activity of PglX that is toxic in *S. coelicolor* and the antitoxin, PglZ, inhibits this. Although we have shown that PglX has methyltransferase activity *in vitro* we were not able to demonstrate the presence of methylated DNA from progeny phage or from M145 (Pgl^+^) undergoing an infection using antibodies against DNA containing N^6^-methyladenine. Recently a phage defence system from *Bacillus cereus* called bacteriophage exclusion or BREX, has been described, that is mediated in part by a homologue of PglX (Goldfarb et al., 2014). In the BREX system the target for the methyltransferase encoded by the *B. cereus* PglX homologue was elucidated by PacBio sequencing. The target, TAGGAG (the underlined A is methylated), is modified in uninfected cells, but infecting phage DNA was not modified despite containing multiple target sites. In the BREX system phage replication is prevented in the first infection, apparently through cessation of phage DNA replication. Thus although BREX and the Pgl systems are mediated by a core of orthologous proteins (PglX, PglY and PglZ in *S. coelicolor* and PglX, BrxC and PglZ in *B. cereus*) it seems there are significant differences in their mechanisms of resistance. We hypothesise that Pgl is adapted to cause phage resistance in the context of a host in which there is no detectable DNA methylation (using antibodies to DNA containing either C^5^-methylcytosine or N^6^-methyladenine; data not shown) and in which there appears to be general methyl-specific restriction (Gonzalez-Ceron et al., 2009; MacNeil, 1988). In this context host DNA might be unmethylated during growth of uninfected host cells and the Pgl system would be inherently toxic unless the methyltransferase activity is highly regulated. We propose therefore that any DNA methylation occurs only during phage infection. The ATPase activity of PglY was also shown to be required for phage resistance, possibly implying the need for a motor to drive a processive activity, similar to Type I R–M systems.

### A model for the mechanism of Pgl

**Fig. 7:**
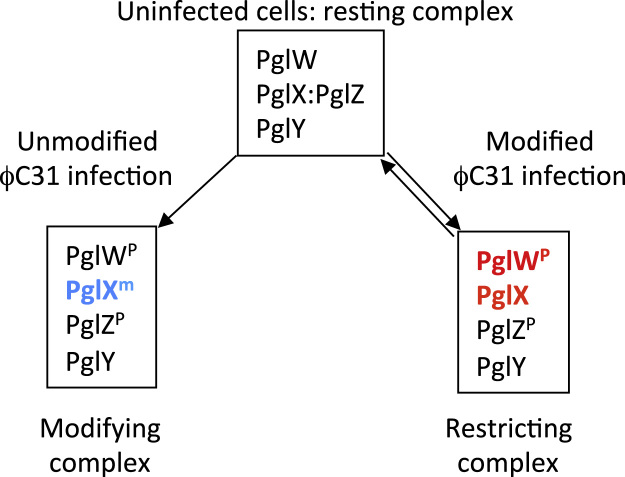
Proposed two step model for the Pgl mechanism by modification and then restriction. In uninfected cells Pgl proteins assemble into a ‘resting complex’ in which the toxic activity of PglX is inhibited by an interaction between PglX and PglZ. Following infection the resting complex changes (presumably triggered by a signal that is specific for phage ϕC31 and its relatives) to become either a DNA modifying complex or a restricting complex, dependent on whether the incoming phage is unmodified or modified, respectively. The modifying complex is likely to have N^6^-adenine methylation activity through the activity of PglX (blue). We propose that the restricting complex is activated by infection by modified phage. Candidate Pgl proteins that could mediate restriction are indicated in red. PglW^P^ and PglZ^P^ are putatively phosphorylated isoforms due to the kinase activity of PglW that transduce a signal to the cells of phage infection (more details are provided in the text).

The model proposed by Chinenova et al. (1982) suggested that Pgl would differ from all known methyl-specific restriction systems owing to the proposed marking of phage or phage DNA and flagging it for restriction in later rounds of infection. We propose a model as to how the activities of the Pgl proteins could mediate the Pgl phenotype *in vivo* (Fig. 7).

The model resembles a Type I R–M system in which the modification and restriction activities are governed by the modification status of the DNA (Dryden et al., 2001; Murray, 2002). We propose that the Pgl proteins switch between three activity states: resting, modifying and restricting. In uninfected cells we propose that the Pgl proteins are in a ‘resting complex’ in which PglZ suppresses the toxic activities of PglX (Fig. 7). Evidence that the Pgl proteins are transcribed was obtained in previous work and PglZ was detected in the *S. coelicolor* proteome (Bedford et al., 1995; Hesketh et al., 2002; Sumby and Smith, 2002). In the model infection by phage coming from a Pgl^−^ host causes a change in the activities of Pgl proteins to modify progeny phage, most likely by N^6^-adenine methylation through the activity of PglX (Fig. 7). The putative N^6^-adenine methyltransferase activity could modify all the DNA in the infected cell or just targets in ϕC31 and its relatives. The trigger for switching between resting and modifying activities of the Pgl proteins might also be specific to infection by ϕC31 and its relatives. To avoid restriction of the modified phage DNA in the first infection cycle, we propose that Pgl proteins in the modifying state cannot switch directly to a restricting state or be reversed back to the resting state. We also propose that host methyl-specific restriction enzymes either do not recognise the modification in progeny phage or that the modification occurs late in the phage replication cycle. Thus in agreement with the observations of Chinenova et al. (1982), modified phage progeny are released in a normal burst. Infection of Pgl^+^ strains with this modified phage triggers the activation of the restricting activity of Pgl (Fig. 7). The mechanism of restriction is not clear but could be mediated by the PglW NERD domain, the host cell methyl-specific restriction endonucleases or an unidentified motif in PglX that is responsible for conferring toxicity. If the latter is the case, PglX could resemble some Type IIS restriction/modification systems that are contained within a single polypeptide (*e.g. Bpm*I and *Eco*57I; Pingoud et al., 2005). We propose that PglW and PglZ sense the phage infection and/or presence of modified or unmodified phage DNA and control the activity of PglX and other Pgl proteins. As PglW has kinase activity and as PglZ have been identified in two isoforms in a proteomics experiment, we propose that control of the Pgl activity state is dependent on phosphorylation (Fig. 5; Bentley et al., 2002; Hesketh et al., 2002; Lomovskaya et al., 1980; Pingoud et al., 2005).

It is known that bacteriophage are an important force for driving bacterial evolution (Weinbauer and Rassoulzadegan, 2004), and the evolution of several types of phage resistance mechanisms, important for avoiding infection and lysis (R–M systems, Abi systems, CRISPR-Cas) suggests evading the deleterious effects of phage is highly selective (Makarova et al., 2013). Data presented here indicate that Pgl is a complex R–M-like system that demonstrates yet further biological novelty in the cellular mechanisms of defence against bacteriophages.

## Materials and methods

### Bacterial strains, plasmids, growth conditions and conjugal transfer from *E. coli* to *Streptomyces*

The *S. coelicolor* strains used in this study are summarised in Table 1. All strains were cultivated on mannitol and soya flour (MS) agar (Hobbs et al., 1989). Plaque assays were performed as in Kieser et al. (2000) on Difco nutrient agar. Bacteriophage ϕC31 *c*Δ25 was used throughout this work as described in Kieser et al. (2000). Plasmids were conjugated into *Streptomyces* from the *E. coli* strain ET12567 (*dam*^*−*^ *dcm*^*−*^ *hsdS*) containing pUZ8002 to provide the transfer functions (MacNeil, 1988). Plasmids used are summarised in Table 2. Plasmids for expression of the His-tagged Pgl proteins in *E. coli* were made as follows starting with the PglX–His_6_ expression plasmid, pPS5032: A 5772 bp BamHI fragment encoding PglX from pPS1001 (Sumby and Smith, 2002) was inserted into BamHI cut pARO191 (Parke, 1990) to generate pPS2002. An NdeI site was introduced at the *pglX* start codon by replacing a 482 bp HindIII–FseI fragment with a 408 bp PCR fragment generate using primers MMUTF and MMUTR and cut with HindIII and FseI to generate pPS3009. DNA encoding the His_6_ tag was added to the 3′ end of *pglX* by replacing a 46 bp AatII fragment with a PCR fragment cut with AatII generated from primers IF2 new and MENDSQ to form pPS5028. The ORF encoding PglX–His_6_ was then cut out from pPS5028 with the NdeI and BamHI sites and the fragment was inserted into NdeI–BamHI cut pT7-7 to form pPS5032. The PglZ–His_6_ expression plasmid, pPS5045 was made as follows: A 5269 bp SstI–BsiWI fragment from pPS5001 (pGem7 containing a 8166 bp NruI fragment encoding *pglYZ* from cosmid 4G2 inserted into the SmaI site; Sumby and Smith, 2002) was replaced with a 218 bp SstI–BsiWI fragment generated by PCR with primers ZNT and ZHisR to introduce an NdeI site at the start of *pglZ*, creating pPS5042. A HindIII site was then introduced just before the stop codon in *pglZ* by replacing a 282 bp SphI fragment with a PCR fragment from primers ZCHisF and ZCHisR, generating plasmid pPS5043. The NsiI–HindIII fragment was then inserted into pPSCHis fusing the 3′ end of *pglZ* to DNA encoding an in frame His_6_-tag, forming pPS5045. (pPSCHis was constructed from pGEM7 by insertion of a 51 bp DNA fragment encoding a His_6_-tag flanked by HindIII and EcoRI sites). The PglW–His_6_ expression plasmid, pPS5012, was constructed as follows: The 1 kbp XbaI–NotI fragment from pPS1001 (Sumby and Smith, 2002) was replaced with a PCR fragment generated using primers KtipF and KtipR resulting in an NdeI overlapping the start of the *pglW* ORF, generating pPS3005. A DNA fragment encoding the His_6_-tag was then added at the 3′ end by replacing the BamHI–EcoRI fragment in pPS3005 with a PCR fragment made using primers KiHis/IRT3 and generating pPS5007. The NdeI–EcoRI fragment from pPS5007 was then subcloned into pT7-7 to generate pPS5012. pPS5025 containing the optimised codons at the start of the *pglW* ORF was created by replacing the NdeI–NotI fragment with a PCR product, generated using primers KCODON and KiseqR, also cut with NdeI–NotI. The His_6_–PglY expression plasmid was generated as follows: pPS5070, encoding an N-terminal His-tag fused to a truncated PglY, was constructed by inserting a PCR fragment, generated using primers YHisF and YHisR and then digested with AatII and SphI, into pGEM7. The entire *pglY* ORF frame was then reconstructed in pPS5071 by inserting the SphI–PvuII fragment from pPS5060 (Sumby and Smith, 2002) into SphI/SmaI-cut pPS5070. The *His*_6_*–pglY* gene was then inserted into pT7-7 using the NdeI–BamHI restriction sites. The pIJ6902-derived, *pglZ*-containing integrating vector, pPH1001, was constructed by subcloning the *Nde*I/*Eco*RI fragment from pPS5045. Primers for PCR reactions are listed in Table S1.

### Disruption of Pgl genes and marker removal in *Streptomyces*

The *pglX* and *pglZ* null mutants were created according to the protocol of Gust et al. (2003) using the pIJ774 apramycin resistance cassette, flanked with *loxP* sites. In the double mutants (Table 1) the marker was removed as described (Khodakaramian et al., 2006) prior to disruption of the second target gene. Oligonucleotides for creating the disruptions are listed in Table S1.

### Site-directed mutagenesis

Point mutants in the Pgl proteins were created using site-directed mutagenesis, performed using the QuikChange XL site-directed mutagenesis kit (Stratagene): primer sequences are detailed in Table S1. The exception to this protocol was in *pglW*, where the parental vector (pPS8002) was too large for Quickchange. The PglW K677A allele was produced by asymmetric PCR of a 900 bp region using the primers in Table S2. A first round of PCR created a product containing the mutant allele, with a flanking natural StuI site (*pglW* K677A F and *pglW Asym FlankR*), and a second PCR created the mutant allele with a flanking SrfI site (*pglW* K677A R and *pglW Asym FlankF*). The products were then mixed and a final round of PCR using the outer PCR primers (pglW Asym FlankF and pglW Asym FlankR) resulted in a final product of 900 bp representing a region of *pglW* with the desired mutation. This fragment was cloned into pGEM-T-Easy, and the mutation confirmed by sequencing. The resulting plasmid, pPH1004, was cut with SrfI and StuI, giving a 460 bp product, which was used to replace the natural fragment in pPS8002, creating the vector pPH1012 (*pglW* K677A).

### Overproduction of Pgl proteins

Pgl proteins were overproduced as His_6_-tagged fusions. The plasmids were introduced in to *E. coli* Rosetta (DE3) (Novagen) and protein expression induced by the addition of 0.1 M IPTG (isopropyl-β-d-thiogalactopyranoside) in exponentially growing cells (optical density 0.5 at 600 nm). The resulting His-tagged proteins were purified by nickel affinity chromatography, and their identity confirmed by MALDI–TOF mass spectrometry.

The exception to this was PglW, which could not be produced in any DE3 lysogens. The protein was expressed from the plasmid pPS5025 using the EcoPro T7 coupled *in vitro* transcription-translation system (Merck Biosciences), with the mass of the expressed protein confirmed by incorporation of ^35^S-methionine in to the translation product according to the manufacturers instructions.

### *In vitro* kinase assay

*In vitro* phosphorylation of PglW was carried out according to Molle et al. (2003). Briefly, PglW was incubated for 1 h at 30 °C in a 20 μl reaction containing 25 mM Tris–HCl, pH7.0, 1 mM DTT, 5 mM MgCl_2_, 1 mM EDTA, 2 μg protein and 200 μCi ml^−1^ [γ-^32^P] ATP. The reaction was stopped by the addition of an equal volume of 2× sample buffer, followed by heating at 98 °C for 5 min. The protein was visualised by autoradiography following separation on 4–12% SDS PAGE gels (Invitrogen).

### ATPase assays

The ability of wild-type and mutant PglY proteins to hydrolyse nucleotides was assayed at 30 °C in 40 mM HEPES·HCl (pH 8.0), 10 mM MgCl_2_, 10 mM dithiothreitol, and 0.1 mg ml^−1^ bovine serum albumin. Nucleotides and protein was added at the indicated concentrations. Reactions (10 µl) were pre-incubated at 30 °C for 10 min with PglY prior to the addition of the nucleotide. Reactions were stopped by the addition of 2.5 µl of stop buffer (100 mM Tris·HCl (pH 7.5), 5% SDS, 200 mM EDTA, and 10 mg ml^−1^ proteinase K) and incubation at 37 °C for 20 min. The hydrolysis of ATP was detected by measurement of the release of inorganic phosphate using acidic ammonium molybdate and malachite green according to the method of McGlynn et al. (2000).

### *In vitro* methylation assays

The reaction mixture contained 50 mM Tris–HCl (pH7.5), 7 mM 2-mercaptoethanol, 1 mM EDTA. 12 μM [^3^H]-*S-*adenosyl-l-methionine (GE Healthcare; specific activity 84 Ci mmol^−1^), 2 μg ϕC31 DNA and 2 μg protein. Reactions were incubated for 1 h at 30 °C. Protein and unincorporated label were removed by phenol extraction and ethanol precipitation. Labelled was detected by liquid scintillation counting.

## Figures and Tables

**Table 1:** *Streptomyces* strains and plasmids used in this study.

Strain	Genotype	Pgl status	References
M145	Prototrophic	+	Kieser et al. (2000)
J1934	Insertional mutation of PglZ	−	Bedford et al. (1995)
SLMW	Δ*pglW* M145 isogenic	−	Khodakaramian et al. (2006)
SPHX	Δ*pglX* M145 isogenic	−	This work
SLMY	Δ*pglY* M145 isogenic	−	Khodakaramian et al. (2006)
SPHZ	Δ*pglZ* M145 isogenic G-tract mutant	−	This work
SPHWZ	Δ*pglWZ* M145 isogenic G-tract mutant	−	This work
SPHXZ	Δ*pglXZ* M145 isogenic G-tract mutant	−	This work
SPHYZ	Δ*pglYZ* M145 isogenic G-tract mutant	−	This work
			
Plasmid	Description		Reference
pIJ6902	Integrating vector with thiostrepton-inducible promoter		Huang et al. (2005)
pT7-7	*E. coli* expression plasmid		Tabor and Richardson (1985)
pPS8002	pglW–His_6_ in pIJ6902		Sumby and Smith (2002)
pPS8003	pglX–His_6_ in pIJ6902		Sumby and Smith (2002)
pPS8008	His_6_–pglY in pIJ6902		Sumby and Smith (2002)
pPH1001	pglZ–His_6_ in pIJ6902		This work
pPH1002	pglX^Y381A^–His_6_ in pIJ6902		This work
pPH1003	His_6_–pglY^K81A,S82A^ in pIJ6902		This work
pPH1005	*pglZ*^D535A^*–His*_*6*_ in pT7-7		This work
pPH1006	pglZ^D694A^–His_6_ in pT7-7		This work
pPH1007	*pglZ*^D535A^*–His*_*6*_ in pIJ6902		This work
pPH1008	pglZ^D694A^–His_6_ in pIJ6902		This work
pPH1010	His_6_–pglY^K81A,S82A^ in pT7-7		This work
pPH1012	pglW^K677A^–His_6_ in pIJ6902		This work
pPS5032	pglX–His_6_ in pT7-7		This work
pPS5045	pglZ–His_6_ in pT7-7		This work
pPS5012	pglW–His_6_ in pT7-7		This work
pPS5025	pPS5012 with optimised codons in *pglW*		This work
pPS5072	*His*_*6*_*–pglY* in pT7-7		This work

**Table 2:** Conjugation efficiency in various strains upon introduction of the PglZ deletion cosmid.

**Strain**	**Genotype**	**Conjugation efficiency** per recipient spore
M145	Parent	1.5×10^−8^
SLMW	Δ*pglW* M145 isogenic	2×10^−7^
SPHX	Δ*pglX* M145 isogenic	2×10^−4^
SLMY	Δ*pglY* M145 isogenic	2×10^−7^
M145 (pPH1001)	Parent, carrying an integrated ectopic copy of *pglZ–His6*	2×10^−4^
M145 (pPH1008)	Parent, carrying an integrated ectopic copy of mutant *pglZ*^*D694A*^*–His6*	3×10^−7^
