# Supplementary material for: The phage growth limitation system in Streptomyces coelicolor A(3)2 is a toxin/antitoxin system, comprising enzymes with DNA methyltransferase, protein kinase and ATPase activity
Source: Virology. 2015 Mar;477:100–9. doi: 10.1016/j.virol.2014.12.036 (PMC4365076; doi:10.1016/j.virol.2014.12.036)
Supplement: Supplementary file 1 — Supplementary data [file mmc1.doc]

# Table S1. Oligonucleotides used in this study

| Name | Sequence |  |
| --- | --- | --- |
| LJM7 | 5’CGAGGTTGTGGCAAGTGGTGTCGAGGGAGACGCGAAGTGATTCCGGGGATCCGTCGACC | Redirect *pglX* forward |
| LJM8 | 5’TGGCCCCTGAACCGACCGACGTCGATCTACTCCTGACTATGTAGGCTGGAGCTGCTTC | Redirect *pglX* reverse |
| LJM3 | 5’CGAGGCCACCATCGAGATCACCTGGCGGGTCGTCGAATGATTCCGGGGATCCGTCGACC | Redirect *pglZ* forward |
| LJM4 | 5’TCAAACTCAAGGACCGAGGCACCCCGAGCCGTGTCCTCATGTAGGCTGGAGCTGCTTC | Redirect *pglZ* reverse |
| MMUTF | 5’AGAAGCTTCACATATGATCGACCGCAAGGCTCTGT | Introduction of NdeI site at *pglX* start codon (SCIF2 27,975-27995) |
| MMUTR | 5’CAGCAAGCGGCCGGCCTGCCCATC | Used with MMUTF (SCIF2 28,381-28,358) |
| IF2New | 5’GCGACGTCGGATCCTAGTGGTGGTGGTGGTGGTGGCTGCCGCGCGGCACCAGCTGCTTCGCTGCTGCGCGAC | 6 x His tag on PglX |
| MENDSQ | 5’ACGTACTTCACCAACACCGC | Used with IF2New (SC4G2 890-909) |
| ZNT | CGGAGCTCCATATGACGGACACCACTGTCGC | Introduces NdeI site at start of *pglZ* |
| ZHisR | GTTCGTGCTGCTCCCGGTCG | Used with ZNT (SC4G2 11,438-11419) |
| ZCHisF | 5’CGCTCACCACCCGCTGGAC | Used with ZCHisR |
| ZCHisR | 5’CGGCATGCAAGCTTGCCCGCTCCGAGGGCGAACTG | Introduces HindIII site at 3’ end *pglZ* |
| KtipF | 5’ATTCTAGACCATATGCGGGAAGGCCGGTGGGT | Introduces NdeI site at start of *pglW* (SCIF2 23,304-23,323) |
| KtipR | 5’CGGCGGCCGCCGGGGGTGGTCT | Used with KtipF (SCIF2 23,572-23,551) |
| KiHis | 5’GCGAATTCTAGACTAGTGGTGGTGGTGGTGGTGGCTGCCGCGCGGCACAAGCTTCGCGTCTCCCTCGACAC | 6 x His tag on PglW |
| IRT3 | 5’CGGGCTTCGAGCTGTCGCTG | Used with KIHis (SC4G2 27,205-27,224) |
| KCODON | 5’CGTCTAGACATATGCGTGAAGGTCGTTGGGTTACTGTTACCGAATCTGAATTCGAACACGAACGTCGTGGCCTGGAGGCGATCCGCCAG | Introduces optimal codons at 5’end *pglW* |
| KIseqR | 5’CTTGGCGCCGGGCTGGGCGA | Used with KCODON |
| YHisF | 5’GCGACGTCATATGCGCGGAAGCCACCACCACCACCACCACGGACTGGTGCCGCGCGGCAGCATGGCCCAGCCGCCCCTCC | 6 x His at N-terminus of PglY |
| YHisR | 5’CCGGAACCGAACGAGCCGTG | Used with YHisF (SC4G2 7,480-7,461) |
| pglW K677A F | 5’-GCACCGGGACATCGCCCCCGACAACATCG-3’ | Introduce K677A mutation in *pglW* |
| pglW K677A R | 5’-CGATGTTGTCGGGGGCGATGTCCCGGTGC-3’ |  |
| pglW Asym FlankF | 5’-[ACGACGCCGACGCCTA](http://bibiserv.techfak.uni-bielefeld.de/cgi-bin/gf_visualize?qid=_1117028494_22607&item=7&FP=176&RP=107)-3’ | Asymmetric PCR over K677A in *pglW* |
| pglW Asym FlankR | 5’- GTGCAGGGCCTTCTGGAA-3’ |  |
| pglX Y381A F | 5'-GGGAATCCGCCG**GCC**ATCACGGTCAAGGA-3' | Introduce Y381A into *pglX* |
| pglX Y381A R | 5'-TCCTTGACCGTGAT**GGC**CGGCGGATTCCC-3' |  |
| pglY K81A/S82A F | 5'-TCGTTCGGTTCCGGT**GCC**GCCCACTTCATGGCC-3' | Introduce K81A,S82A into *pglY* |
| pglY K81A/S82A R | 5'-GGCCATGAAGTGGGC**GGC**ACCGGAACCGAACGA-3' |  |
| pglZ D535A F | 5’-GATGCTCGTGCTCGCCGGCATGAGCGCGG-3’ | Introduce D535A into *pglZ* |
| pglZ D535A R | 5’-CCGCGCTCATGCCGGCGAGCACGAGCATC-3’ |  |
| pglZ D694A F | 5’-CGTCCTCACCAGCGCCCACGGCCACGTCG-3’ | Introduce D694A into *pglZ* |
| pglZ D694A R | 5’-CGACGTGGCCGTGGGCGCTGGTGAGGACG-3’ |  |
